# A Multiplicative Approach to Polyvictimization: A Study of Intimate Partner Violence Types as Risk Factors for Child Polyvictimization in South Korea

**DOI:** 10.3390/ijerph16050783

**Published:** 2019-03-04

**Authors:** Clifton R. Emery, Hyerin Yang, Oksoo Kim, Yoonjeong Ko

**Affiliations:** 1 SWSA, University of Hong Kong, Hong Kong, China; 2 Transitional Justice Working Group, Seoul 03142, Korea; hyerin87@naver.com; 3 Yonsei University School of Social Welfare, Seoul 139-720, Korea; mydreamunicef@gmail.com (O.K.); koyoonjeong@nate.com (Y.K.)

**Keywords:** IPV typology, child polyvictimization

## Abstract

Drawing on a new typology of intimate partner violence (IPV), this paper tests the relationship between indicators of totalitarian and anarchic IPV and child polyvictimization incidence and severity. The paper argues for and utilizes a quantitative approach to study polyvictimization severity. Polyvictimization is operationalized as a multiplicative relationship between physical abuse and neglect in a random sample of 204 children from Kyunggi province, South Korea. The indicator of totalitarian IPV significantly predicted polyvictimization severity and incidence even when a traditional measure of intimate terrorism was held constant. The indicator of anarchic IPV significantly predicted polyvictimization severity but not incidence when a traditional measure of intimate terrorism was held constant. Implications are discussed.

## 1. Introduction

Research on harm stemming from children’s exposure to multiple forms of victimization, conceptualized as polyvictimization, is relatively new in the research literature [1,2]. Not only does polyvictimization appear to be more harmful to children than single victimization, but low-frequency victimization across multiple domains (e.g., bullying at school and abuse at home) appears to produce more distress than high-frequency victimization in a single domain [1]. Child polyvictimization is associated with more psychopathology, depression, delinquency, low self-esteem, self-blame, suicidal phenomena, health risk behaviors, and anxiety among children, and higher unemployment, substance abuse and mental illness among parents [1,3,4,5,6,7]. Finkelhor et al. argued that there are four pathways to child polyvictimization: a) dangerous families, b) problem-beset families, c) dangerous communities, and d) children with pre-existing emotional problems that increase risky behaviour [1]. Chan extended the concept to incorporate co-occurrence of multiple forms of violence at the family level, labelling this family polyvictimization [2]. Family polyvictimization was indicated by child victimization, intimate partner violence (IPV), and elder abuse, and was associated with parental addiction and poor health in a Chinese household sample [2]. Despite Chan’s initiative, polyvictimization remains understudied in an East Asian context. Moreover, research on the dangerous families pathway [1] generally focuses on individual characteristics of family members rather than family systemic characteristics, little work has been done to link the concept of polyvictimization with its measurement, and the problem of how to incorporate high frequency single-victimization with polyvictimization has not been resolved. Specifically, despite a large literature on the co-occurrence of IPV and child maltreatment [8], little literature has examined the effects of types of IPV on child polyvictimization. Similarly, no existing measure integrates polyvictimization incidence and frequency into a common scale of polyvictimization severity. This paper examines the relationship between polyvictimization and a new typology of violent family organization in a probability proportional to size random cluster sample of rural South Korean children. We hypothesize anarchic and totalitarian family types are at higher risk for polyvictimization. The paper argues for and implements a measure of polyvictimization that allows single-victimization frequency and polyvictimization to be combined in the same scale that captures the severity of polyvictimization. We argue this measure is closer to the conceptual rationale for the study of polyvictimization.

Although Chan [2] has stressed the importance of considering emergent qualities of the family as a unit in considering polyvictimization, and a 30-year research tradition documents conceptual and empirical links between intimate partner violence and child maltreatment [8,9,10,11], only a handful of studies searchable via google scholar explicitly examine intimate partner violence and child polyvictimization. Chan’s research examined exposure to parental IPV as one component of polyvictimization and found that polyvictimized children had lower self-esteem, higher rates of aggression, PTSD, addiction, and lower quality of life [2,5,12]. Pereda and Gallardo-Pujol similarly included IPV exposure as polyvictimization and found that it predicted re-victimization in adulthood [13]. Using a similar concept of polyvictimization, other research found that the relationship between child polyvictimization and PTSD were mediated by child attributions [14] and that polyvictimization was correlated with later disordered eating [15]. 

### 1.1. Disorder vs. Deviant Order: Totalitarian and Anarchic Family Types as Risk Factors for Polyvictimization

Although the foregoing literature on child polyvictimization has considered IPV exposure as a form of polyvictimization [5], it has insufficiently examined parental IPV in an etiological role with respect to polyvictimization via the dangerous families pathway [1], neither has it conceptualized IPV as having a profound effect on emergent family processes. Emery [16] argues that the introduction of physical violence into an intimate relationship shifts the foundation on which power rests. According to this argument, power dynamics between intimate partners in never-violent relationships rest on walk-away costs. However, when an act of physical violence occurs between partners the base of power may shift from walk-away costs to force. In that case power may rapidly shift to the partner who can command the most physical force [16], permanently changing family power dynamics. In Emery’s typology, IPV exists on dual continua of order and power, ranging from anarchic type (low order, chaotic, no consistent rules or legitimate power) at one extreme and totalitarian type (highly ordered, asymmetric power) at the other [16]. Totalitarian families are characterized by power asymmetry and elaborate systems of rules extending control to “mundane areas of everyday life …not normally thought of as norm- or rule-governed” [17]. Emery argues that the anarchic type is chaotic, unpredictable, and fits more with the social disorganization conceptualization of IPV common in criminology, while the totalitarian type reflects the traditional feminist conceptualization of IPV. IPV at both extremes, however, is likely to create a more dangerous family environment for children [16]. IPV at the extremes of these continua can put children at higher risk not only because the IPV may be more frequent and qualitatively severe [16], but also because maltreatment may be more likely to be legitimized as punishment for rule-breaking in the totalitarian type while family rules (norms) aimed at protecting children may be absent in the anarchic type. This suggests families with any history of IPV and characterized by anarchy or totalitarian style control may put children at particular risk for polyvictimization.

Although some research [18,19] has begun to use the concept of the totalitarian type, no research has used a measurement specifically aimed at capturing anarchic or totalitarian type families, and no research has examined these types as risk factors for polyvictimization. Indeed, research on types of IPV in an etiological role for child polyvictimization broadly is lacking. Although arguably the most common typology of IPV is Johnson’s [20] intimate terrorism typology, a google scholar search of “intimate terrorism” and “polyvictimization” returns only 47 results, none of which feature intimate terrorism in a causal role for child polyvictimization. The intimate terrorism typology is defined on the basis of control motive [20], with the result that whether control attempts succeed or not (achieved control) is ignored. The intimate terrorism typology has been critiqued on this basis as ignoring power, for which reason this paper focuses on the anarchic/totalitarian IPV typology in etiological relation to child polyvictimization.

### 1.2. Rationale for Polyvictimization and Its Measurement: Capturing Severity

The burgeoning literature on polyvictimization [1,2,3,4,5,6,7,8,9,10,11,12,13,14,15] rests on a largely implicit rationale: polyvictimization must be distinct in both etiology and impact from high frequency victimization of a single type. If this were not the case, multiple experiences of victimization of different forms could lumped together additively in a single measure without reference to type. For example, experience of acts of physical abuse, acts of neglect, and witnessing acts of IPV could simply be summed. The rationale for polyvictimization as a unique subject is empirically supported by findings that even low-frequency polyvictimization appears to have a more severe impact than high frequency single-victimization [1]. The problem for the polyvictimization field to date [1,2,3,4,5,6,7,8,9,10,11,12,13,14,15] is that researchers are forced to choose between examining the impact of frequency for single-victimization or sums of dichotomous indicators for various types of single-victimization that add up to polyvictimization. Continuous measures generally have more statistical sensitivity than dichotomous measures, and Emery et al. [21,22] have long argued for and implemented continuous measures of violent victimization weighted by the log-odds of injury. Although the continuous measure creates a right-skewed distribution, this problem can now easily be handled via monotonic transformation, robust standard errors, or both [23]. Moreover, a continuous measure can capture the severity, rather than simply the fact, of polyvictimization.

We argue that the empirical logic of the rationale for the study of polyvictimization is one of interaction effects. That low frequency victimization in the context of a second form of victimization has a larger impact on child well-being than high frequency single-victimization [1] suggests that the effect of one form of victimization on well-being differs depending on whether a second form is present or absent. Interaction effects capture just such an effect, as when the effect of witnessing IPV on externalizing behavior problems depends on the child’s age [24]. The standard approach to handle this problem in linear models is to model multiplicative relationships between variables, (e.g., age X witnessing IPV) rather than only additive relationships [23,24]. In this paper, we present a continuous measure of polyvictimization which is additive when a single form of victimization is present but multiplicative when multiple forms are present. This extends the scale to higher levels when more than one kind of victimization is present. For example, if our measure of physical abuse severity is a 5 and only physical abuse is present, then the continuous polyvictimization scale will also be a 5. However, if the child has a physical abuse score of 5 and also a neglect score of 2, then the polyvictimization score will be 2 × 5 = 10. Our models compare the continuous severity measure with a more standard categorical measure of polyvictimization.

### 1.3. Current Study

Based on the IPV literature, two hypotheses were made:

**Hypothesis** **1.** *Polyvictimization and polyvictimization severity will be positively associated with totalitarian type IPV.*

**Hypothesis** **2.** *Polyvictimization and polyvictimization severity will be positively associated with anarchic type IPV.*

The aim of the current study was to extend the literature on polyvictimization by examining whether emergent family types (anarchic and totalitarian) from the IPV literature [16] are related to polyvictimization and comparing a new severity measure of polyvictimization with the standard approach. The current study also contributes to the IPV literature. The full models control for a measure typically used to capture a type of IPV known as intimate terrorism [20]. Intimate terrorism is commonly studied in the IPV literature, and is defined as violence in the context of systematic attempts to control the victim [20]. The intimate terrorism concept has been critiqued as inadvertently ignoring power [16]. If the anarchic and totalitarian family types predict polyvictimization when intimate terrorism is held constant this would suggest some support for the added value of the disorder to deviant order continuum theory (anarchic/totalitarian typology) [16]. Models also control for age and sex of the focal child, sex of the parent, and household income. 

## 2. Methods

### 2.1. Study Design and Sample

Research participants were drawn from a probability proportional to size (PPS) cluster sample of parents of public-school children in Kyunggi province, South Korea. Eight rural public schools were selected using PPS sampling, after which grades were randomly selected via PPS sampling. Once a class within a school was randomly selected, the study team used connections with school officials, teachers, and parents to reach children’s parents, with the goal of obtaining all of the parents of children in the grade selected. Participation was incentivized with gift cards, and a refusal conversion protocol was used to keep the response rate high (81.5%). Refusal conversion attempts to convert a ‘soft no’ into an agreement to participate by using a different interviewer, a different time of approach, and a different incentive. When parents had more than one minor child, they were asked to select the child with the most recent birthday as the focal child. The total sample consisted of 107 girls and 128 boys with a mean age of 11.7 years old (SD = 4.2, *n* = 235). However, 13 parents were omitted from analyses for skipping or failing to answer correctly a quality control question (“If you read and understand this question circle the 5”). Interviewers underwent two days of rigorous training on written informed consent, sensitivity and study ethics, passing both written and oral exams prior to certification. The study protocol was approved by the IRB of Yonsei University.

### 2.2. Measurements

#### 2.2.1. Polyvictimization

Two scales of polyvictimization were created, both based on underlying data from the Parent-Child Conflict Tactics Scale (PC-CTS) [25]. Responses to all items were once in the last year, twice in the last year, 3–5 times, 6–10 times, 11–20 times, more than 20 times, not in the last year, or never. Physical abuse and neglect of the child were measured by 8 violence items, 4 injury items, and 3 neglect items: (1) hit child on the bottom with a belt, hairbrush, or stick, (2) threw something at him/her, (3) hit some other part of the body with a belt, hairbrush, or stick, (4) pushed, grabbed, or shoved him or her, (5) kicked, bit, or hit him/her with fist, (6) choked or knocked him/her down, (7) beat up him/her, (8) intentionally burned or scalded him/her, (9) kid had a bruise from a conflict with parent, (10) kid had a cut from a fight with parent, (11) kid needed to see a doctor (MD) because of a fight with parent, (12) kid had to miss school because of a fight with parent, (13) had to leave child alone when she/he needed looking after, (14) could not give him/her the food she needed, and (15) child ran away and I did not know where he/she was (α = 0.86). Last year injuries were totaled and regressed on physical violence items, creating regression coefficients that indicated the average increase in injuries for each act of a particular type of violence. These coefficients were used to weight total acts of each type of violence and sum the weighted acts into a scale of physical abuse severity. The benefit of this scale is that an act of a particular type of physical violence is weighted by its associated injuries, avoiding false equivalence between low injury violence like hitting on the bottom with an object and high injury violence like intentional burning. Total last year acts of neglect were summed into a single scale. Prior to combining the two scales were standardized to have the same variance.

Broadly speaking, the continuous polyvictimization measure can be understood as capturing the severity of polyvictimization. The continuous polyvictimization scale was created by setting the scale to zero when there was no abuse or neglect, equal to the physical abuse severity scale when there was no neglect, equal to the number of acts of neglect when there was no physical abuse, and neglect times physical abuse when both were present. A log transform was used to reduce right-skew. The conventional polyvictimization scale was 0 for no victimization, 1 for single victimization, and 2 for both kinds of victimization.

#### 2.2.2. Totalitarian and Anarchic-Type IPV

The totalitarian and anarchic types are principally defined by order (a highly norm- and rule-governed family routine) and power (between intimate partners). These concepts were captured using previously published measures of family order [26] and an updated version of decision power [27]. IPV was measured using the Conflict Tactics Scale 2 (CTS2) [28]. 

Family order was captured by 9 Likert scale items (strongly agree–strongly disagree): In my family (1) we eat dinner together, (2) we eat dinner at the same time every night, (3) our house/apartment is neat and orderly, (4) our house/apartment is clean, (5) I know the daily schedules of everyone in our home, (6) I know what household chores it is my job to do, (7) I know what household chores other people in my family are supposed to do, (8) when one person in the family is sick or really busy other family members step in to do that person’s chores, (9) our family has many rules (α = 0.79). 

Power between intimate partners was measured using answers to who had more say in decisions about (1) buying a car, (2) buying a computer, (3) children’s education, (4) buying a house/apartment, and (5) where to live. Choices ranged on a scale from 1–5 (spouse only to self only) with 3 indicating self and spouse equally (α = 0.70). Equal power was coded ranging from 0% to 100% depending on how many items for which respondents indicated a ‘3’. 

Last year IPV items were answered for self and spouse, and consisted of (1) slapped, (2) hit partner with something, (3) had to see a doctor because of a fight with partner, (4) punched, kicked or bit, (5) used or threatened to use a knife or gun, (6) had a sprain, bruise, small cut, or felt pain the next day because of a fight with partner, (7) pushed, grabbed or shoved, (8) beat up, (9) choked (α = 0.97). Possible responses are the same as for the PC-CTS. Any IPV was indicated if any of these items had ever occurred by male or female partner. 

Relatively few families were characterized by low order, so low order was defined as below the median. This helps to meet the minimum theoretical characteristics of the anarchic type while ensuring a reasonable number of families are included in the category. Families low in order with ever any IPV were classified as anarchic type. High family order was defined as above the 75th percentile. Totalitarian type was classified if there was ever any IPV, family order was high, and equality in power was less than or equal to 83%. Similar to the case for anarchic type, this cut-off meets the minimum characteristics for the distinctly totalitarian type while ensuring a reasonable number of families meet the criteria. 

#### 2.2.3. Intimate Terrorism

In theory, intimate terrorism is characterized by violence in the context of a high level of attempted control [20]. The original research on intimate terrorism operationalized the concept using multiple scales, one of which was control attempts: thinking about your husband would you say he (1) is jealous or possessive, (2) tries to limit your contact with family and friends, (3) insists on knowing who you are with at all times, (4) calls you names or puts you down in front of others, (5) makes you feel inadequate, (6) shouts or swears at you, (7) prevents you from knowing about or having access to the family income, even when you ask [20,29]. For female participants the questions were left as above and asked on a 5-item Likert scale (1. never – 5. always) (α = 0.87). For male participants, “my partner complains that I…” was added in front of each item so that the scale consistently captures attempts to control the female partner. For the male respondents, a 6th option on the Likert scale “my partner does not complain but I actually do this”. This was coded as the highest value on the scale (6) (α = 0.84). In the original research the items were used to create a dichotomous measure of intimate terrorism [20]. However, we keep a continuous (summed) measure because this allows the measure to have more explanatory power, which raises the empirical bar that the anarchic and totalitarian types must meet for significance. For the same reason, rather than combining control attempts with IPV, any IPV is introduced and controlled separately. The full model controlling for both any IPV and continuous control attempts can be said to control for intimate terrorism. 

### 2.3. Statistical Analyses

The models of the continuous measure of polyvictimization were implemented using mixed effects regression models with a random effect for each cluster and robust standard errors. A Pearson’s goodness of fit test was not significant, (χ^2^ = 147.4, *p* = 0.997), which suggests a Poisson regression (rather than negative binomial) model can be used for categorical polyvictimization. Likewise, a Vuong test (*p* = 0.50) suggests no difference between zero-inflated and an ordinary Poisson model. Hence, Poisson models with robust standard errors were implemented to test the hypotheses using the conventional measure of polyvictimization. Item non-response for physical abuse and neglect narrowed the analysable sample to 215 for the Poisson models. Fewer cases (204) were available for the continuous measure of polyvictimization because a missing response for any abuse or neglect measure resulted in a missing value. For the conventional measure of polyvictimization, item non-response only resulted in a missing value of all other responses to abuse (or neglect) were ‘never’. Both models are presented for the 204 cases available for the continuous model, but the findings for the Poisson models using 215 cases were not noticeably different in sign, significance, or magnitude. Ordinal logits were used to estimate odds-ratios for Figure 2. A priori power analyses were difficult to estimate as this constitutes a first test of these two hypotheses. Post-hoc power analyses suggest the study was sufficiently powered for hypothesis 1 (λ = 0.96, power = 99%) but power to test hypothesis 2 was substantially weaker (λ = 0.45, power = 59%).

## 3. Results

### 3.1. Prevalence and Demographics

**Figure 1:**
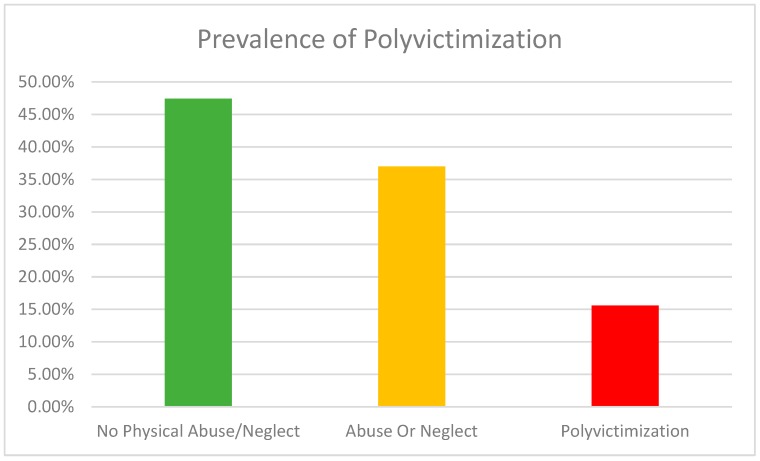
Prevalence of polyvictimization.

Figure 1 shows 15.6% of children (34) in the sample suffered both physical abuse and neglect (polyvictimization) in the last year. 37.0% suffered from either abuse or neglect, but not both. The mean for the continuous polyvictimization measure was -.84 (SD = 1.49). The prevalence of any last-year physical abuse was 38.9% and any last-year neglect was 29.4%. The last year prevalence of very severe abuse (beat up, choked, intentionally burned) was 4.1%; abuse injury was 3.6%. The rate of very severe abuse was not significantly different from the 5.8% rate previous research found in Seoul (Z = 0.89, *p* = 0.37) but the rate of injuries was significantly lower than Seoul’s 8.5% rate (Z = 2.3, *p* = 0.02) [22]. With respect to children in the sample, 11% had experienced insufficient food in the past year; 24.3% had been left alone when parents thought they should not.

Of children in the sample, 12.3% had been exposed to some form of IPV, 3.5% (8) lived in households characterized as totalitarian type, and 4.0% (9) lived in households characterized as anarchic type. The mean score for attempts to control the female partner was 13.4 (SD = 5.6). Previous research has characterized two standard deviations above the mean as ‘high control’ [18]; 4.7% of the sample met this threshold. Although this is not significantly different from a previous study of Korea (5.2%, Z = 0.2, *p* = 0.79) [18], power to detect a difference from the previous study is low for this comparison. There were more female than male caregivers (63.2%) in the sample. The average child’s age was 11.8 (SD = 4.2). There were slightly more male than female (54% vs 46%) children in the sample, and the median household income was 3.5 million won per month (3130 USD).

### 3.2. Association between Polyvictimization and Anarchic and Totalitarian Types

**Figure 2:**
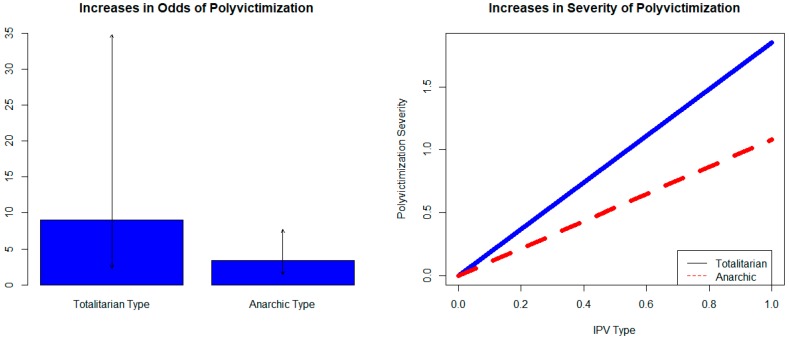
IPV type and polyvictimization.

Figure 2 shows increases in odds of polyvictimization associated with anarchic and totalitarian types (left side) from baseline ordinal logit models and increases in severity of polyvictimization (right side) from baseline mixed effects regression models. The baseline models control for child’s age, sex, participant sex, and household income. Figure 2 shows that when totalitarian type IPV is present odds of polyvictimization are 9 times higher, and when anarchic type is present odds of polyvictimization are 3.3 times higher. Arrows indicate 95% confidence intervals around odds-ratios. The right side of Figure 2 shows that totalitarian type is significantly associated with a 1.85 (1.2 standard deviation) increase on the polyvictimization severity scale, and anarchic type is significantly associated with a 1.08 (0.7 standard deviation) increase on the severity scale.

### 3.3. Association between Polyvictimization and Anarchic and Totalitarian Types Controlling for Intimate Terrorism

Table 1 shows the results of Poisson regression for conventional polyvictimization (left side) and mixed effects regression models for polyvictimization severity (right side). The table allows readers to compare the baseline models with models controlling for attempts to control the female partner and any IPV. The final model controls for attempts to control the female partner and any IPV, and hence controls for intimate terrorism. In the final model, totalitarian type IPV is significantly associated with a 0.77 increase on the polyvictimization categorical scale (*p* < 0.001). Likewise, totalitarian type IPV is associated with a 1.8 unit increase in polyvictimization severity (*p* < 0.001). In the same model, anarchic type IPV is no longer significantly associated with polyvictimization categorically, but remains significantly associated with a 0.78 unit increase in polyvictimization severity (*p* < 0.01). 

## 4. Discussion

### 4.1. Anarchic and Totalitarian IPV as Risk Factors for Child Polyvictimization

This is the first study to operationalize the theoretical concepts of anarchic and totalitarian IPV and examine their relationship to child polyvictimization. As hypothesized, both totalitarian and anarchic types of IPV were significantly associated with the incidence and severity of child polyvictimization in models controlling for child’s age, sex, parent’s sex, and household income. The significant findings suggest that polyvictimization is both more likely and more severe when IPV occurs in the context of a highly ordered family with an extensive (and perhaps intrusive) set of rules for governing behavior and in which the couple is unequal in power. The findings also suggest that child polyvictimization is more likely and more severe when IPV occurs in chaotic families characterized by low order.

The study simultaneously adds to our understanding of IPV. The relationship between child polyvictimization and totalitarian type IPV persisted even when intimate terrorism was controlled. Emery [16] argued that the classification of IPV by control attempts alone [20] was a theoretical liability. Rather, he argued that IPV must be classified based on underlying continua of order, power, and legitimacy. Our findings suggest that further investigation of this argument may bear empirical fruit. More research is needed to establish whether Emery’s [16] anarchic/totalitarian typology of IPV is consistently related to polyvictimization and other child well-being outcomes. This research should continue to compare that typology [16] with the effects of Johnson’s [20] intimate terrorism typology of IPV. There are many similarities between these two typologies. However, if the former continues to show stronger empirical relationships with child well-being than the latter, researchers on child well-being may eventually wish to adopt the typology with more explanatory power. Furthermore, if anarchic and totalitarian types place children at greater risk, policy makers may be able to efficiently mitigate risk by targeting interventions specifically at families with these types of IPV.

### 4.2. Further Research Needed on Polyvictimization Severity

This study is the first to use a measure of child polyvictimization severity as well as incidence. The severity scale was created by accounting for the effects of individual acts of physical abuse on injuries, the frequency of neglect, and the assumption of interaction effects between different types of victimization. The polyvictimization severity scale highlights the theoretical salience of polyvictimization and prior research: victimization across different domains may be worse for children than more victimization in a single domain [1]. As hypothesized, findings for the polyvictimization severity scale were similar to findings for the conventional polyvictimization incidence measure. As expected, there is evidence to believe the severity scale to be more statistically sensitive (anarchic type IPV remained a significant predictor of polyvictimization severity but not polyvictimization incidence when intimate terrorism was controlled). These findings suggest polyvictimization severity is worth further study.

Further research is needed to develop the measure of polyvictimization severity. More types of child victimization could be included in such a measure in future. Moreover, conceptual and empirical work is needed to establish logical impact measures on which to base polyvictimization severity. The contribution of the perpetration of a particular act of violence to the increase in log odds of victim injury is a relatively logical criterion for severity of physical violence. Although this paper examined IPV at any time, severity of exposure to IPV could also be measured in terms of how much each act of IPV increased the likelihood of child injury (accidental or deliberate). However, reasonable indicators of neglect severity are more complex. Malnutrition might be a suitable outcome as it would catch problems stemming from both insufficient food and child consumption of unhealthy food due to lack of monitoring. However, determining the answer to this problem lies beyond the scope of this paper.

### 4.3. Child Victimization Rates Are High in Regional Context

The study found that more than 1 in 6 children in this rural sample of South Korean children had experienced polyvictimization in the previous year. More than half of the sample (52.6%) had experienced either physical abuse or neglect in the past year. This rate of child victimization is a reliable prevalence estimate based on a random sample of families with children in rural Kyunggi province. It is significantly higher (*p* < 0.01) than the 42.8% child victimization rate found in a representative sample of 7466 Chinese households [2] found in previous research. This finding suggests that child protection in rural South Korea is at least as serious an issue as in China, if not more so.

### 4.4. Limitations

Although the study is a representative random sample, the sample is small, and findings may not be generalizable beyond rural South Korean children. The measures of anarchic and totalitarian types of IPV are preliminary and the cut-off points should be replicated and examined in a larger study. Because the study is cross-sectional and non-experimental, statistical inferences are indicators of associations that are not necessarily causal. The findings are preliminary and require replication, and the child polyvictimization severity scale requires further conceptual and empirical development. Although anarchic type IPV was consistently associated with polyvictimization severity, it was not significantly associated with the categorical polyvictimization when intimate terrorism was controlled. This null finding may have occurred because there is no true relationship or because of the relatively small sample size. Only one parent was interviewed per family, and many types of polyvictimization (exposure to bullying and street crime) were not included.

## 5. Conclusions

As the first study of the relationship between totalitarian and anarchic types of IPV and child polyvictimization the findings have important implications for understanding risk factors for polyvictimization. Important progress has been made in reducing IPV related homicide by using Domestic Violence High Risk Teams (DVHRTs) to assess for high risk cases and follow up with additional resources and increased monitoring [30]. These targeted intervention programs for IPV could be extended to monitor the risk for child polyvictimization. Future research is needed to replicate and extend these findings in diverse and larger samples. Polyvictimization poses a serious and unique threat to children. Should future research confirm our preliminary findings, policy makers should provide funds to pilot the extension of DVHRTs to monitor for child polyvictimization. Research is also needed to better understand the effects of totalitarian and anarchic types of IPV on victims, and particularly how these effects differ from or converge with the effects of intimate terrorism. The initial results suggest the anarchic/totalitarian IPV typology may be at least as effective (if not more so) as the intimate terrorism typology for understanding the relationship between high risk IPV and child polyvictimization. The mental, physical, and emotional costs of child polyvictimization are high [1,2,5,12]. We owe it to our children to continue to try to do better.

## Figures and Tables

**Table 1:** Polyvictimization incidence and severity (*n* = 204).

Variable	Polyvictimization Incidence			Polyvictimization Severity		
	*B*	*B*	*B*	*B*	*B*	*B*
Totalitarian Type	0.82 ***	0.84 ***	0.77 ***	1.85 ***	1.88 ***	1.81 ***
Anarchic Type	0.55 **	0.39 ^†^	0.28	1.08 ***	0.89 ***	0.78 **
Child’s Age	−0.02	−0.02	−0.02	0.01	0.01	0.01
Child’s Sex (Female)	0.28 *	0.31 *	0.30 *	0.36 ^†^	0.38 ^†^	0.37
Respondent Sex	0.20	0.23	0.22	0.28	0.30	0.29
Household Income (100,000 KRW)	0.004	0.004	0.004 ^†^	0.005	0.005	0.005
Attempted Control of Female Partner		0.02	0.02		0.02	0.02
Any Intimate Partner Violence			0.15			0.16

*Notes*: ^†^*p* < 0.10, * *p* < 0.05, ** *p* < 0.01, *** *p* < 0.001.
